# Liver histopathological alteration and dysfunction after bisphenol A administration in male rats and protective effects of naringin

**DOI:** 10.22038/AJP.2021.17649

**Published:** 2021

**Authors:** Masoud Mahdavinia, Layasadat Khorsandi, Soheila Alboghobeish, Azin Samimi, Mohammad Amin Dehghani, Leila Zeidooni

**Affiliations:** 1 *Toxicology Research Center, Ahvaz Jundishapur University of Medical Sciences, Ahvaz, Iran*; 2 *Cellular and Molecular Research Center, Ahvaz Jundishapur University of Medical Sciences, Ahvaz, Iran*; 3 *Department of Pharmacology, School of Pharmacy, Student Research Committee, Ahvaz Jundishapur University of Medical Sciences, Ahvaz, Iran*; 4 *Department of Toxicology, School of Pharmacy, Student Research Committee, Ahvaz Jundishapur University of Medical Sciences, Ahvaz, Iran*

**Keywords:** Bisphenol A, Naringin, Liver histopathological alteration, Oxidative stress, Rat

## Abstract

**Objective:**

Bisphenol A (BPA) is an organic synthetic compound, often used in manufacturing polycarbonate plastics. Researches have shown the role of BPA as an endocrine disruptor. The present study intended to evaluate the hepatoprotective properties of naringin, an active flavanone glycoside present in many citrus fruit, against hepatotoxicity induced by BPA.

**Materials and Methods:**

Male Wistar rats were orally treated with 50 mg/kg BPA for 30 consecutive days for induction of toxicity and 40, 80 and 160 mg/kg naringin for the same period along with BPA or alone.

**Results:**

This study demonstrated that BPA significantly increased serum levels of triglyceride, lactate dehydrogenase (LDH), alkaline phosphatase (ALP), lipid peroxidation, and aspartate aminotransferase (AST) and significantly reduced catalase, glutathione peroxidase (GPx) and superoxide dismutase (SOD) activity, glutathione (GSH) and caused periportal inflammation and microvesicular steatosis in rat tissue. However, BPA did not change serum levels of high-density lipoprotein-cholesterol (HDL-C), total cholesterol, alanine aminotransferase (ALT), or low-density lipoprotein-cholesterol (LDL-C). Furthermore, the results displayed that administration of 80 and 160 mg/kg naringin improved hepatotoxicity and altered lipid peroxidation level, serum values of triglyceride and liver enzymes, and oxidative stress factors that were induced by BPA. The effect of two doses of 80 and 160 mg/kg naringin was more noticeable than that of dose 40 mg/kg.

**Conclusion:**

The findings suggested the protective effects of naringin against BPA-induced hepatotoxicity via ameliorating liver histopathological alteration, suppressing oxidative stress and lipid-lowering properties.

## Introduction

Bisphenol A (BPA), as an endocrine disruptor, is commonly used in production of epoxy resins, polycarbonates, dental sealants, food packages, baby bottles and mineral water containers (Soares et al., 2009 ▶). Numerous studies have showed water and food contamination with BPA. Thus, water and food consumption can be considered an important route of exposure (Mikołajewska et al., 2015 ▶).

Contact with acid or basic compounds and heat accelerates the hydrolysis of polycarbonate bonds and epoxy resins in BPA molecules, and leads to BPA entering food and beverages (Mikołajewska et al., 2015 ▶). BPA due to its phenolic structure similar to diethylstilbestrol (DES), reacts with estrogen receptors but because of weaker estrogenic characteristics than DES, has a dual behavior of agonist and, in some cases, antagonist for endocrine receptors (Snyder et al., 2000 ▶).

Studies have proven endocrine disorders induced by BPA such as polycystic ovarian syndrome, infertility, and precocious puberty (Mikołajewska et al., 2015 ▶). Earlier reports have indicated the side effects of BPA in the reproduction system, immune system and nervous system in human and animal (Santangeli et al., 2017 ▶). Obesity, diabetes, cardiovascular disorders and cancer can be attributed to BPA (Gong and Han, 2006 ▶; Nakamura et al., 2010 ▶). BPA exposure has recently been reported to have adverse effects on liver function (Sun et al., 2020 ▶). 

The liver is the first and most important organ in which BPA metabolism occurs. Consequently, the liver can be more vulnerable to low BPA doses than other organs. BPA is generally metabolized by the CYP2C cytochrome family in the liver in two pathways (Niwa et al., 2001 ▶); in the major pathway, BPA is metabolized and eliminated by combining with a glucuronide and/or a sulfate. Alternative pathway includes corrosion by hydroxylation to a catechol and then alteration to an o-quinone. The catechol-o-quinone formed is the main cause of interference in the redox cycle along with reactive oxygen species (ROS) formation. Under physiological conditions, o-quinones have a high affinity for electrons and easily transmit electrons. Even very few quantities of o-quinone are enough to produce large levels of oxidative stress (Kovacic, 2010 ▶). 

Various studies confirmed the side effects of BPA on liver function. BPA has been revealed to be able to increase liver enzymes, oxidative stress, lipid peroxidation following inducing inflammation and mitochondrial dysfunction (Bindhumol et al., 2003 ▶, Elswefy et al., 2016 ▶), increase insulin resistance (Geng et al., 2017 ▶), and induce hepatosteatosis (Martella et al., 2016 ▶) and progression of hepatic tumors (Weinhouse et al., 2014 ▶) in liver of experimental animals and human.

**Figure 1:**
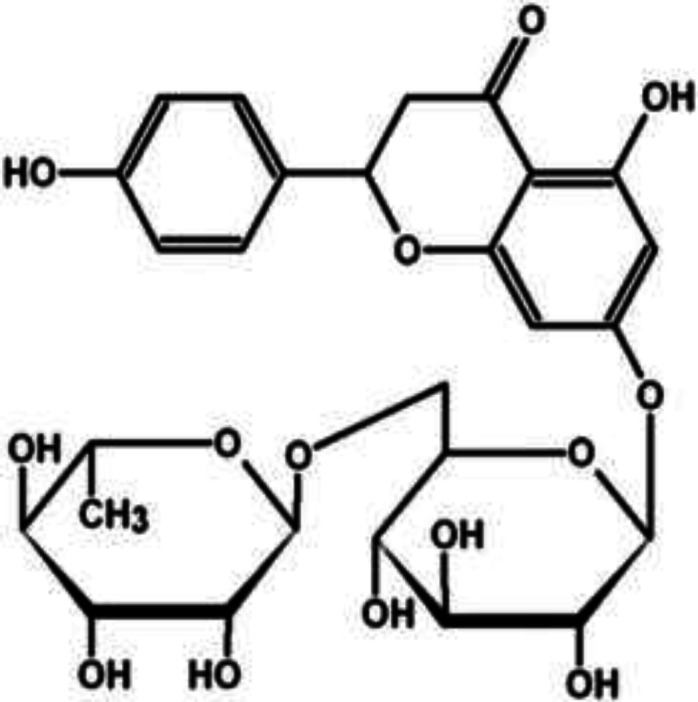
Structure of naringin (Kwatra et al., 2016 ▶).

Naringin (4', 5, 7-trihydroxy flavanone 7-rhamnoglucoside) is a main flavanone glycoside of many citrus fruit (Figure 1). Naringin was recently indicated to have antioxidant, free radical scavenging, antiinflammatory (Kandhare et al., 2014 ▶), neuroprotective (Gopinath et al., 2011 ▶), nephroprotective (Badary et al., 2005 ▶), antihypertensive, anti-apoptotic, wound-healing (Kandhare et al., 2016 ▶), cholesterol-lowering (Jeon et al., 2004 ▶), antimutagenic and anticancer properties (So et al., 2009 ▶).

Therefore, this study was designed to assess the protective effect of naringin against BPA-induced hepatotoxicity and oxidative stress in rats.

## Materials and Methods

**Chemicals**

BPA (99% pure), naringin (95% pure), trichloroacetic acid (TCA), thiobarbituric acid (TBA) and reduced glutathione were acquired from Sigma-Aldrich (St Louis, Missouri, USA) and 5, 5’-dithiobis-2-nitrobenzoic acid (DTNB) was achieved from Merck (Darmstadt, Germany). The rest of the chemicals were acquired from the highest and best commercial grade.

**Animals**

In this study, adult male Wistar rats (six-week old, weighing 200-215 g) were prepared. Rats were kept in polypropylene cages with 12 hours of light and dark cycles, at room temperature 22±2°C and 10% humidity and they received water and standard pellet diet *ad libitum*. Rats were obtained from the animal center of Ahvaz Jundishapur University of Medical Science (AJUMS) and the study was approved by Ethical Committee Acts of AJUMS (IR.AJUMS.REC.1396.293) for Care and Use of Laboratory Animals.

**Experimental design**

The rats were randomly grouped into six groups (n = 6). 

Group 1: As control group, rats orally received 0.5 ml/animal of olive oil.

Group 2: As BPA group alone, rats orally received BPA (50 mg/kg) (Hassani et al., 2017 ▶).

Groups 3, 4 and 5: Rats orally received BPA (50 mg/kg) plus 40, 80 and 160 mg/kg of naringin, respectively (Khodayar el al., 2020 ▶).

Group 6: Rats orally received naringin 160 mg/kg alone. 

BPA was dissolved in olive oil and given daily for 30 days. Twenty-four hour after the final treatment, the animals were anesthetized by ketamine-xylazine (40 mg/kg - 5 mg/kg). The samples of blood were immediately collected from retro-orbital puncture by using sterile polished micro capillary tubes and serum was separated for biochemical analysis. The livers also were collected and homogenized with phosphate buffer (pH 7.4) in a ratio of 1 to 10 (w/v) for oxidative stress analysis.

**Body weight, and absolute and relative liver weight measurement**

Animals were weighed on the first and last day of the study, then sacrificed, and their livers were fast dissected and weighed**. **To calculate the relative weight of the liver, first the liver weight of each rat was divided by the final body weight and reported as percentage (Khodayar et al., 2020 ▶).

**Biochemical assays**

Assay kits for evaluation of aspartate aminotransferase (AST), alanine aminotransferase (ALT), lactate dehydrogenase (LDH) and alkaline phosphatase (ALP) as well as low-density lipoprotein (LDL), high-density lipoproteins (HDL), triglyceride (TG), and total cholesterol were used (Pars Azmoon Kit. IRI).

**Assessment of oxidative stress**

Superoxide dismutase (SOD), glutathione peroxidase (GPx), catalase (CAT), glutathione (GSH) and malondialdehyde (MDA) evaluation was performed according to the standard protocols prepared by assay kits of ZellBio Company (Hinter den Gärten, Lonsee, Germany).

**Histopathological evaluation of the liver**

The rat livers were fixed in 10% formalin solution for 24 hr, then embedded in paraffin. Then, 5 µm sections were stained with hematoxylin and eosin (H&E). Seven slides were reviewed for tissue alterations assessment in terms of congestion of RBCs, infiltration of inflammatory cells, sinusoidal dilation and fat deposits, by using an optical microscope. Then, all tissue changes observed were categorized.

**Statistical analysis**

Data were examined by one-way ANOVA test followed by Tukey’s *post hoc* test for comparison among groups. Results are expressed as mean±SEM (n=6). Data analysis was completed using the Prism 5.0 (San Diego, CA, USA) statistical package program. A value of p<0.05 was considered to be statistically significant.

## Results

**Treatment with BPA and naringin did not change body and liver weight**

As Table 1 shows, no significant difference in the initial body weight was observed among the groups. Also, after 30 days of BPA exposure no significant effect on the final body weight, or absolute and relative liver weight of the animals in comparison with the control group was observed. No significant difference was observed in the groups treated with naringin on the mentioned factors in comparison with the control or the BPA groups.

**Treatment with BPA and naringin changes the activity of liver enzymes**

As Table 2 shows, no significant difference was observed in serum levels of ALT between the BPA and control group. AST, ALP and LDH activity significantly augmented in the BPA group in comparison with the control group (p<0.01 and p<0.001, respectively). However, in all groups treated with naringin plus BPA a significant decrease in AST (BPA + naringin 40 mg/kg p<0.05, BPA + naringin 80 and 160 mg/kg p<0.01) and LDH (all three doses plus naringin p<0.001) activity in comparison with the group that received BPA was observed. Naringin 160 mg/kg plus BPA meaningfully diminished ALP activity in comparison with the BPA group alone (p<0.01). 

**Treatment with BPA and naringin changes the lipid profile**

As Table 3 shows, there was only a noteworthy enhance in TG level in the BPA group in comparison with the control group. Groups treated with naringin at 80 and 160 mg/kg showed a significant reduce in TG levels that were induced by BPA (p<0.01). 

**Treatment with naringin ameliorates antioxidants activity**

Table 4 shows antioxidants activity in the experimental groups. The BPA-treated group alone showed a significant reduction in SOD, CAT and GPX activity in rat liver in comparison with the control group (p<0.001). Treatment with naringin at 80 and 160 mg/kg significantly improved SOD (both doses p<0.05), CAT (BPA + naringin 80 mg/kg p<0.01 and BPA + naringin 160 mg/kg p<0.001) and GPX (BPA + naringin 80 mg/kg p<0.05 and BPA + naringin 160 mg/kg p<0.001) activities in comparison with the BPA group alone.

**Table 1:** The effect of naringin against BPA toxicity on body weight and liver weight on animals. Findings are shown as mean±SEM (n=6).

Groupings Variables	Control	BPA 50 mg/kg	BPA + Nar 40 mg/kg	BPA + Nar 80 mg/kg	BPA + Nar 160 mg/kg	Nar 160 mg/kg
Initial bod weight (g)	203±1.04	205±2.02	200±1.20	210±2.10	206±2.14	214±1.21
Final body weight (g)	233.8±3.94	234.8±3.46	225.2±4.60	225.4±1.72	221.4±2.59	230.2±2.05
Absolute heart weight (g)	8.53±0.721	8.89±0.524	8.46±0.424	7.78±0.420	8.52±0.325	8.94±0.230
Relative liver weight *100	3.64±0.05	3.86±0.06	3.75±0.18	3.45±0.24	3.84±0.31	3.88±0.15

Bisphenol A (BPA), naringin (Nar).

**Table 2:** The effect of naringin and bisphenol A (BPA) on liver enzymes of rats. Results are mean±SEM (n=6).

Groupings Variables	Control	BPA 50 mg/kg	BPA + Nar 40 mg/kg	BPA + Nar 80 mg/kg	BPA + Nar 160 mg/kg	Nar 160 mg/kg
ALT (U/L)	61.6±1.9	75.4±2.1	56.67±3.2	54.6±2.5	65.5±3.2	51.42±4.3
AST (U/L)	111.8±8.1	201.6±10.8**	141.3±4.5**#**	131.7±3.4**##**	123.7±4.7**##**	116.5±3.2
ALP (U/L)	573.2±37.7	981.3±25.4*	880.3±6.6	663.5±28.2	596.8±52.2**##**	562.5±43.3
LDH (U/L)	783.5±50.12	2247.6±76.15**	942.8±40.18**###**	794.3±33.64**###**	750.52±26.71**###**	735.3±28.42

Alanine aminotransferase (ALT), aspartate aminotransferase (AST), alkaline phosphatase (ALP), and lactate dehydrogenase (LDH), bisphenol A (BPA), naringin (Nar).

**p<0.001 and *p<0.01 significant differences between the BPA group and the control group.

#p<0.05, ##p<0.01 and ##p<0.001 significant differences among naringin-treated groups against the bisphenol A (BPA) group.

**Table 3:** The effect of naringin and bisphenol A (BPA) on lipid profile of rats. Results are mean±SEM (n=6).

Groupings Variables	Control	BPA 50 mg/kg	BPA + Nar 40 mg/kg	BPA + Nar 80 mg/kg	BPA + Nar 160 mg/kg	Nar 160 mg/kg
Cholesterol (mg/dl)	60.2±2.4	57.6±3.1	63.2±1.78	61.2±4.2	61.7±2.1	66.3±2.6
Triglyceride (mg/dl)	44.6±3.4	89.5±5.4*	65.3±3.4	58.3±5.4**#**	52.2±5.4**#**	49.5±3.4
LDL-C (mg/dl)	30.2±1.2	32.1±2.1	31.2±3.1	29.3±2.4	28.3±1.9	27.1±1.1
HDL-C (mg/dl)	27.2±2.1	31.4±3.1	34.4±1.8	36.6±3.1	38.5±3.2	35.2±4.2

Low-density lipoprotein-cholesterol (LDL-C), and high-density lipoprotein-cholesterol (HDL-C), Bisphenol A (BPA), naringin (Nar).

*p<0.001 significant differences between the BPA group and the control group.

#p<0.05 significant differences between the naringin-treated groups and the BPA group.

**Table 4:** The effects of naringin and bisphenol A (BPA) on the antioxidant enzymes. Results are mean±SEM (n=6).

Groupings Variables	Control	BPA 50 mg/kg	BPA + Nar 40 mg/kg	BPA + Nar 80 mg/kg	BPA + Nar 160 mg/kg	Nar 160 mg/kg
SOD (U/ mg protein)	7.60±0.52	3.74±0.67**	5.45±0.38	6.16±0.38**#**	6.47±0.54**#**	7.54±0.48
GPX (U/ mg protein)	7.048±0.52	2.56±0.32**	4.030±0.27**	4.636±0.27*#	5.66±0.41**###**	6.936±0.57
CAT (U/ mg protein)	157.9±9.14	72.6±5.4**	93.45±3.45**	110.54±7.8**##	128.40±3.9*###	149.54±6.14

Superoxide dismutase (SOD), catalase (CAT), and glutathione peroxidase (GPx), bisphenol A (BPA), naringin (Nar).

**p<0.001 and *p<0.05 significant differences between the BPA group and the control group.

#p<0.05, ##p<0.01, and ###p<0.001 significant differences between the naringin-treated groups and the BPA group.

**Treatment with naringin increases GSH level**

**Figure 2:**
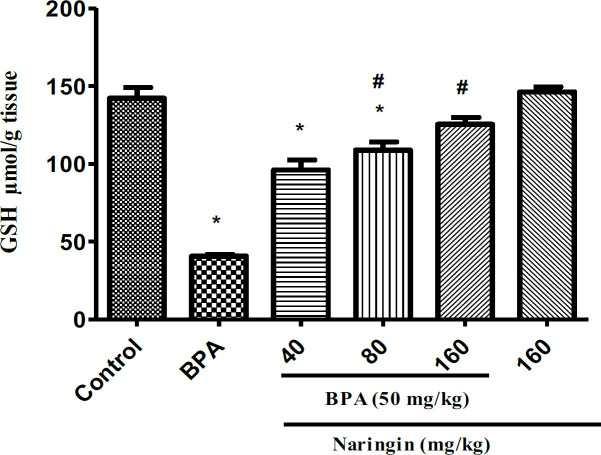
The effect of naringin and bisphenol A (BPA) on the glutathione (GSH) amounts in rat's liver. Results are shown as mean±SEM (n=6).

As Figure 2 shows, there was a significant reduction in liver GSH levels in the BPA group in comparison with the control group (p<0.001). However, treatment with naringin at 80 (p<0.05) and 160 (p<0.05) mg/kg significantly augmented liver GSH levels in comparison with the BPA group. 

**Treatment with naringin decreases MDA level**

**Figure 3:**
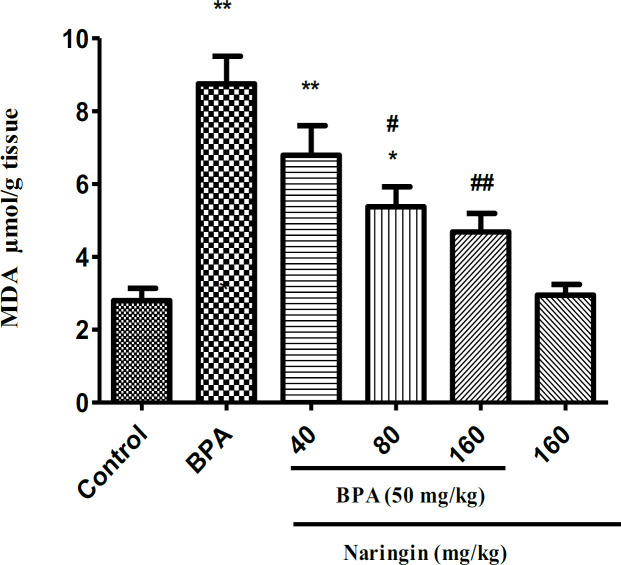
The effect of naringin and bisphenol A (BPA) on the malondialdehyde (MDA) amounts in liver of rat. Data are shown as mean±SEM (n=6).

As Figure 3 shows there was a significant increase in the MDA levels in the BPA group alone in comparison with the control group (p<0.001). However, treatment with naringin 80 and 160 mg/kg significantly reduced MDA levels in comparison with the BPA group alone (p<0.01 and p<0.001 respectively).

*p<0.001 significant differences between the BPA group and the control group.

#p<0.05 significant differences between the naringin-treated groups and the BPA group.

*p<0.05 and **p<0.001 significant differences between the BPA group and the control group.

#p<0.05 and ##p<0.01 significant differences between the naringin treatment groups and the BPA group.

**Figure 4:**
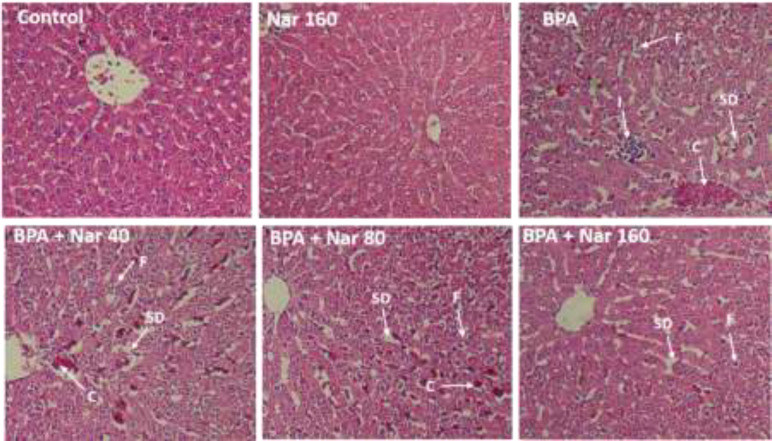
Images of the liver observed by optical microscopy and stained with H&E (magnification 400X). Nar (naringin), BPA (bisphenol A).

SD: Sinusoidal dilation; I: Infiltration of inflammatory cells; F: Fat deposit and C: Congestion of RBCs.

**Morphological changes of liver tissue**

Figure 4 and Table 5 show liver tissue alterations in the experimental groups. The control and naringin 160 mg/kg alone groups had a normal tissue Accumulation of erythrocytes, microvesicular steatosis and inflammation were observed in the BPA group. However, treatment with naringin 80 and 160 mg/kg ameliorated the mentioned changes.

**Table 5:** Quantification of damage in rat liver tissue

Groupings Variables	Control	BPA 50 mg/kg	BPA + Nar 40 mg/kg	BPA + Nar 80 mg/kg	BPA + Nar 160 mg/kg	Nar 160 mg/kg
Congestion of RBC	0.0	1.7 ± 0.14**	1.4 ±0.22**	0.9 ±0.15**##	0.2 ±0.01**###	0.0
Infiltration of inflammatory cells	0.0	1.4 ±0.13**	0.7 ±0.09**#	0.1±0.06**###	0.06±0.002*###	0.0
Fat deposit (%)	0.0	20.7±3.1**	18.3±0.23**	8.7 ±1.2**##	3.7 ±0.4**###	0.0
Sinusoidal dilation	0.02±0.002	2.2±0.35**	1.8±0.21**	0.9±0.02**#	0.2±0.04**###	0.01±0.003

Data are shown as mean ± SEM. Bisphenol A (BPA), naringin (Nar).

*p<0.01, and **p<0.001 significant differences between the BPA group and the control group.

#p<0.05, ##p<0.01, and ###p<0.001 significant differences between the naringin-treated and the BPA group.

**Figure 5:**
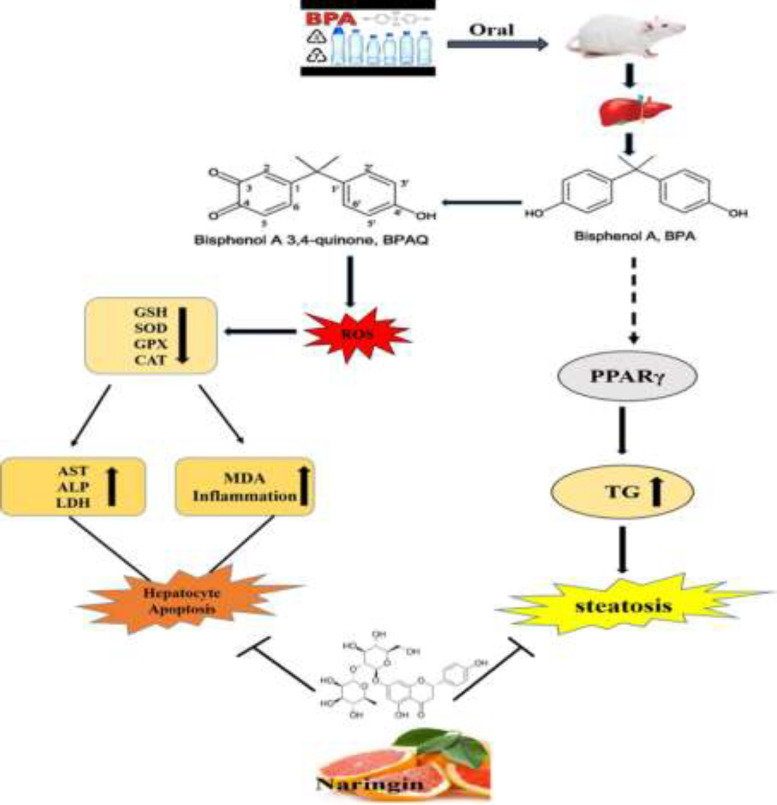
Hepatotoxicity caused by BPA and hepatoprotective effects of naringin

## Discussion

BPA is an endocrine disturbing chemical released in the environment. So far, few reports have shown BPA effects on the liver while many studies has been done for toxic effect on reproductive system (Haavisto et al., 2003 ▶; Maffini et al., 2006 ▶). For investigation of hepatoprotective properties of naringin on BPA-induced toxicity, we measured body and liver weight, serum biochemical indices, and oxidative stress markers and assessed histopathology changes after 30 days of treatment with 50 mg/kg BPA and 40, 80 and 160 mg/kg naringin, in rats. We observed a significant enhancement in AST, ALP, LDH, TG, and MDA levels and diminution in GSH amount, and SOD, CAT and GPX activity as well as microvesicular steatosis and periportal inflammation in BPA-treated rats.

In our study, BPA had no effect on weight gain in rats. In human studies, the relationship between obesity and BPA exposure is one of the most contradictory and argued issues. Various studies have shown a statistically significant relationship between the urinary and serum levels of BPA with weight gain, and other studies have found no connection between these two (Metwally et al., 2016 ▶). In experimental studies, there is a contradiction similar to human populations. Some studies revealed that BPA was correlated with weight gain in rats after 30 days. However, some studies similar to this study showed that there was no association between BPA and weight gain (Hassani et al., 2017 ▶).

In our pervious study, similar to this study, BPA-treated rats did not show any weight gain after one month of treatment (Khodayar et al., 2020 ▶). It can be concluded that several risk factors are involved in the development of obesity. Therefore, in this study, which was a one-month treatment period, it was difficult to find a connection between BPA intake and weight gain in rats.

 Liver enzymes ALT, AST, ALP and LDH are released into the bloodstream following inflammation and necrosis and are reliable biomarkers for indicating liver injury (Giannini et al., 2005 ▶). The proinflammatory cytokines including interleukin-1β (IL-1β), IL- 6 and tumor necrosis factor-alpha (TNF-α) are made by hepatocytes following liver damage. These cytokines are released from the liver and they can activate macrophages during injury and cause inflammation. It was shown that BPA exposure up-regulated the mRNA levels of liver pro-inflammatory cytokines including, IL-1β, IL- 6 and TNF-α in mice liver and caused liver injury (Hussein and Eid, 2013 ▶). Our findings showed that BPA exposure at 50 mg/kg for 30 days, caused liver damage including, periportal inflammation and augmented serum levels of LDH, ALP and AST. In this study, treatment with 40, 80 and 160 mg/kg naringin and 50 mg/kg/day BPA ameliorated histopathological changes induced by BPA including periportal inflammation and significantly improved the serum of AST, ALP and LDH activity. The effect of the doses of 80 and 160 mg/kg of naringin was more noticeable than that of dose 40 mg/kg. In agreement with our study, it was observed that naringin 20, 40 and 80 mg/kg improved hepatotoxicity caused by nickel sulfate through ameliorating histopathological lesions and reduction of serum levels of AST, ALT, ALP, LDH, and bilirubin amount and gamma glutamyl transferase activity in rat liver, and 80 mg/kg of naringin was selected as the most effective dose (Pari and Amudha, 2011 ▶). 

BPA alters energy balance, stimulates lipid accumulation and adipogenesis, increases serum triglyceride and cholesterol levels and leads to increased BMI and obesity. Previous studies have reported that BPA exposure for 30 days at 50, 500, and 5000 mg/kg, augmented fatty acid and TG levels (Marmugi et al., 2012 ▶; Moghaddam, 2015 ▶), also rats treated with BPA 0.5, 5 and 50 mg/kg for 30 days showed a significant increase in serum levels of TG whereas there was no change in total cholesterol and LDL-C quantities (Hassani et al., 2017 ▶). In agreement with previous studies, the present study presented that treatment with BPA 50 mg/kg for 30 days elevated TG quantities while did not change cholesterol, LDL-C and HDL-C amounts but caused microvesicular steatosis in the liver tissue. Our results showed that naringin administration significantly ameliorated histopathological changes induced by BPA including microvesicular steatosis and also reduced serum levels of triglyceride. The impact of BPA on dyslipidemia is because of disruption of several hormones such as adiponectin and leptin regulating the energy consumption, alteration of several metabolic functions and destruction of endogenous hormones. BPA binds to estrogen receptor (ER), G- protein-coupled receptor 30 (GPR30) (Thomas and Dong, 2006 ▶), estrogen-related receptor (ERR) (Takayanagi et al., 2006 ▶), peroxisome proliferator-activated receptors (PPARs) and the aryl hydrocarbon receptor (AhR) (Krüger, Long, and Bonefeld-Jørgensen, 2008 ▶) resulting in increased serum triglyceride and cholesterol levels, lipoprotein lipase activity and triacylglycerol accumulation, leading to obesity (Metwally et al., 2016 ▶). 

Oxidative stress reflects an imbalance in the redox cycle, which is characterized by an increase in the production of free radicals and / or ROS and a decrease in antioxidant defense, a process that initiates and promotes liver damage (Xianchu et al., 2016 ▶). Oxidative stress is caused by a multitude of bioactive elements such as quinones and aromatic nitro complexes and conjugated imines which interfere with the redox cycle and result in ROS formation. The monoquinone is the yield of BPA in minor metabolic pathway in the presence of tyrosinase. O-quinone can cause oxidative stress by oxidative enzyme, metal ion, or in some cases molecular oxygen which is the initiator of BPA hepatotoxicity (Kovacic, 2010 ▶).

BPA in the liver increases lipid peroxidation, resulting in cell membrane damage, mitochondrial dysfunction (Moon et al., 2012 ▶), steatosis and steatohepatitis (Huc et al., 2012 ▶). Our findings showed that BPA increased level of MDA while naringin administration at 80 and 160 mg/kg obviously decreased level of MDA. Previous studies revealed that naringin has an anti-lipid peroxidation activity and exerts a protective role against oxidative stress-induced by xenobiotics. This protective effect and the scavenging of free radicals was shown to be due to direct interaction of naringin with ROS through the present of the hydroxyl groups in the naringin molecule (Iwahashi et al., 1990 ▶; Ribeiro et al., 2008 ▶).

Glutathione (GSH) is an important intracellular antioxidant molecule and as a co-factor for GPx enzyme that produces GSSG as a by-product for inactivation of xenobiotic molecules and reactive substances of lipid peroxidation. The sulfhydryl groups of reduced glutathione interact with catechol-o-quinone, leading to its excretion from the body. Therefore, GSH depletion by catechol-o-quinone leads to damages to macromolecules including thiol proteins and membrane lipids (Kwatra et al., 2016 ▶). Data displayed that the GSH amounts were considerably drained by BPA in treated rats, this may have contributed to the expansion of liver damage induced by BPA (Harisa et al., 2014 ▶; Hassani et al., 2017 ▶; Jagetia and Reddy, 2011 ▶).

SOD, CAT and GPX enzymes as indicators of the amount of tissue damage induced by xenobiotics, protect cells against damages caused by oxidative stress. In the present study, BPA-induced liver toxicity resulted in a significant decrease in the activities of enzymatic antioxidants, possibly due to neutralization of free radical produced by BPA in the liver. In concordance with our results, a significant reduction in SOD, CAT and GPX activity induced by BPA at 0.1, 1, 10, 50 mg/kg/day in rat liver, was shown (Hassan et al., 2012 ▶). Previous studies have shown that naringin has antioxidant properties along with upregulating SOD, CAT and GPX genes expression (Jeon et al., 2001 ▶). In our investigation, treatment with naringin 80 and 160 mg/kg augmented activities of SOD, CAT and GPX possibly because of free radical scavenging effects.

Our data show that BPA caused hepatotoxicity via increasing ROS. Previous reports have suggested that one of the reasons underlying ROS formation by BPA is probably activation of MAPK/ERK pathway (Ptak and Gregoraszczuk, 2012 ▶; Ptak et al., 2014 ▶). In this study, naringin was able to improve hepatotoxicity by inhibiting ROS production and enhancing antioxidant defense. A possible mechanism underlying naringin ROS scavenging effect is its inhibitory effect on mitogen-activated protein kinases (MAPK) activation. MAPK are a family of serine/threonine kinases mediators of intracellular signals in response to various stimuli. P38 MAPKs, ERK1/2 and JNK are three different groups of MAPKs in mammals. Previous studies have proven that naringin evidently down-regulated p-p38 MAPK, p-ERK1/2 and p-JNK expression suggesting that naringin may prevent ROS development induced by activation of MAPK pathway. These properties of naringin somewhat might be related to its antioxidant effects (Chen et al., 2014 ▶). 

In summary, this study demonstrated that the administration of naringin protects the liver against BPA induced toxicity in rats. Naringin ameliorated histopathological changes, liver enzymes, lipid profile and antioxidant/oxidative stress indicators of hepatotoxicity. This protective effect of naringin could be because of its antioxidant ability, which led to free radicals scavenging (Figure 5). Therefore, this study suggested that naringin could be an alternative agent to liver therapy. 
